# Early diagnosis and treatment of leptospirosis: Optimizing clinical outcomes

**DOI:** 10.1016/j.jinf.2025.106675

**Published:** 2026-01-10

**Authors:** Umaporn Limothai, Nattachai Srisawat, David A. Haake

**Affiliations:** a Center of Excellence in Critical Care Nephrology, Chulalongkorn University, Bangkok, Thailand; b Excellence Center for Critical Care Nephrology, King Chulalongkorn Memorial Hospital, Bangkok, Thailand; c Division of Nephrology, Department of Medicine, Faculty of Medicine, Chulalongkorn University, Bangkok, Thailand; d Academy of Science, Royal Society of Thailand, Bangkok, Thailand; e Veterans Affairs Greater Los Angeles Healthcare System, Los Angeles, CA, United States of America; f David Geffen School of Medicine at UCLA, Los Angeles, CA, United States of America

**Keywords:** Leptospirosis, Anti-bacterial agents/therapeutic use, Early diagnosis, Time-to-treatment, Prognosis, Risk assessment, Critical illness/prevention & control, Supportive care

## Abstract

Leptospirosis is a globally prevalent zoonotic infection causing more than one million cases and nearly 60,000 deaths annually yet is often diagnosed late after organ dysfunction and other complications have arisen. Delayed diagnosis leads to late initiation of antibiotics and other therapeutic interventions, at which point complications such as renal failure, jaundice, or pulmonary hemorrhage are more common and therapy is less effective. This review highlights the critical importance of early recognition and intervention, emphasizing the therapeutic window during the leptospiremic phase when antibiotics are most effective. We examine the limitations of current clinical and laboratory diagnostic methods, the evolving role of molecular and biomarker-based platforms, and the potential of integrated scoring systems for frontline triage. Evidence supporting early antibiotic therapy, supportive care strategies, and severity prediction tools is summarized. We propose a paradigm shift toward field-adaptable, point-of-care diagnostics and integrated care pathways to ensure earlier treatment, improved outcomes, and reduced global disease burden.

## Introduction

Leptospirosis is a globally prevalent cause of acute undifferentiated febrile illness (AUFI) that poses significant diagnostic challenges in its early stages. Based on a comprehensive systematic review of published studies and national databases from 34 countries, Costa et al. estimated that leptospirosis causes approximately 1.03 million cases and 58,900 deaths each year worldwide.^1^ The global incidence rate was estimated at 14.77 cases per 100,000 population per year, with significantly higher rates in tropical regions—up to 150.7 per 100,000 in some areas.^2^ Mortality rates vary by region and healthcare access, but severe cases, especially those progressing to Weil’s disease or pulmonary hemorrhage syndrome, have fatality rates ranging from 5% to over 50%. Leptospirosis was found to be the most common etiology of AUFI in Southeast Asia in a comprehensive literature review, accounting for 12.1% of cases, followed by dengue and influenza.^3^ In Latin America, the reported incidence of AUFI etiologies varies by region and season depending on rainfall and flooding. However, leptospirosis was the most common AUFI etiology among inpatients^4^ and outpatients^5^ in two prospective studies in Colombia. These numbers place leptospirosis among the most significant zoonotic diseases globally, with a burden comparable to or exceeding that of more well-known diseases such as dengue fever and schistosomiasis.^2^ Yet, leptospirosis remains underdiagnosed and underreported due to limited diagnostic capacity available at points of care in endemic regions.^2^ Importantly, much of the morbidity and mortality is preventable: with early diagnosis and prompt antibiotic treatment, the progression to severe disease and death can often be averted.

The initial symptoms of leptospirosis—typically including fever, muscle pain, and headache—are nonspecific and in the endemic regions where it occurs resemble those of other acute febrile illnesses (AFIs) such as dengue, influenza, and malaria. Given that the incidences of both leptospirosis and dengue increase dramatically with seasonal rainfall and flooding, co-infections with both etiologies are common and associated with high mortality.^6^ This clinical and seasonal overlap often leads to delayed or missed diagnoses, hindering early recognition and therapeutic interventions. Epidemiologic factors provide important clues to timely and accurate diagnosis - the infection is primarily transmitted through contact with water or soil contaminated by the urine of infected animals, particularly rodents, livestock, and domestic pets, which act as reservoir hosts by shedding bacteria from their kidneys. While more common in tropical and subtropical regions, leptospirosis can occur anywhere that humans are exposed to contaminated environments. In the early stages of infection, *Leptospira* rapidly disseminates and multiplies, often reaching high concentrations in the bloodstream before triggering the immune system. Once antibodies are produced and begin to deliver organisms to phagocytic cells, the host response can be severe, potentially involving life-threatening multisystem organ failure impairing the function of the kidneys, liver, lungs, and other organs. Delayed initiation of appropriate management beyond the initial leptospiremic phase is much less likely to be effective in preventing such complications.

There is an urgent need to shift the paradigm of when leptospirosis is diagnosed and managed toward earlier and more effective recognition and interventions. *Leptospira* species are highly susceptible to routine antibiotics and when treatment is provided at an early stage of infection, numerous studies (reviewed below) demonstrate the effectiveness of therapeutic interventions. Currently, healthcare providers typically rely on clinical diagnosis and routine laboratory testing, which have limited sensitivity and specificity for leptospirosis. Standard serologic tests such as IgM ELISA and the Microscopic Agglutination Test (MAT) usually involve shipping samples to central laboratories, where the results are primarily used for epidemiologic purposes. Although point-of-care serologic tests are becoming more widely available, patients with early infection are often seronegative as the antibody response typically occurs toward the end of the first week of infection. While a number of accurate laboratory-based pathogen-specific molecular diagnostic tests are now available, there is a need to invest in transitioning these technologies to point-of-care diagnostic platforms. A closer connection is needed between leptospirosis diagnosis and therapy, in which diagnostics are clearly intended to guide therapeutic decision-making for individual patients at the point-of-care and to facilitate identification of infection at an early stage when treatment can have the greatest impact.

The goals of this review are to define best practices and approaches for achieving early diagnosis and treatment of leptospirosis at a stage of infection when antibiotics can be most effective. We will examine challenges and limitations of early diagnosis with currently available clinical and laboratory approaches. Emerging technologies and platforms for advancing early diagnosis using molecular approaches, as well as cytokine, proteomic, and transcriptomic biomarkers will be explored. Implementation strategies to facilitate early diagnosis and treatment are essential, including creating more awareness among patients and healthcare providers, the role of integrated public health systems, and how to improve access to early diagnostic tools in endemic regions through investment in research and development of low-cost, accurate diagnostic testing. The impact of early therapy on healthcare outcomes will be reviewed, examining evidence for the effectiveness of early antibiotic treatment, roles of early and more aggressive supportive care, and why early treatment is important to minimize complications, including persistent symptoms and chronic illness. By addressing these key aspects, this review aims to highlight the critical need for a shift in clinical and public health strategies to prioritize early, point-of-care diagnostics and timely therapeutic interventions for leptospirosis, ultimately improving patient outcomes and reducing disease burden.

## Background

**Fig. 1.**
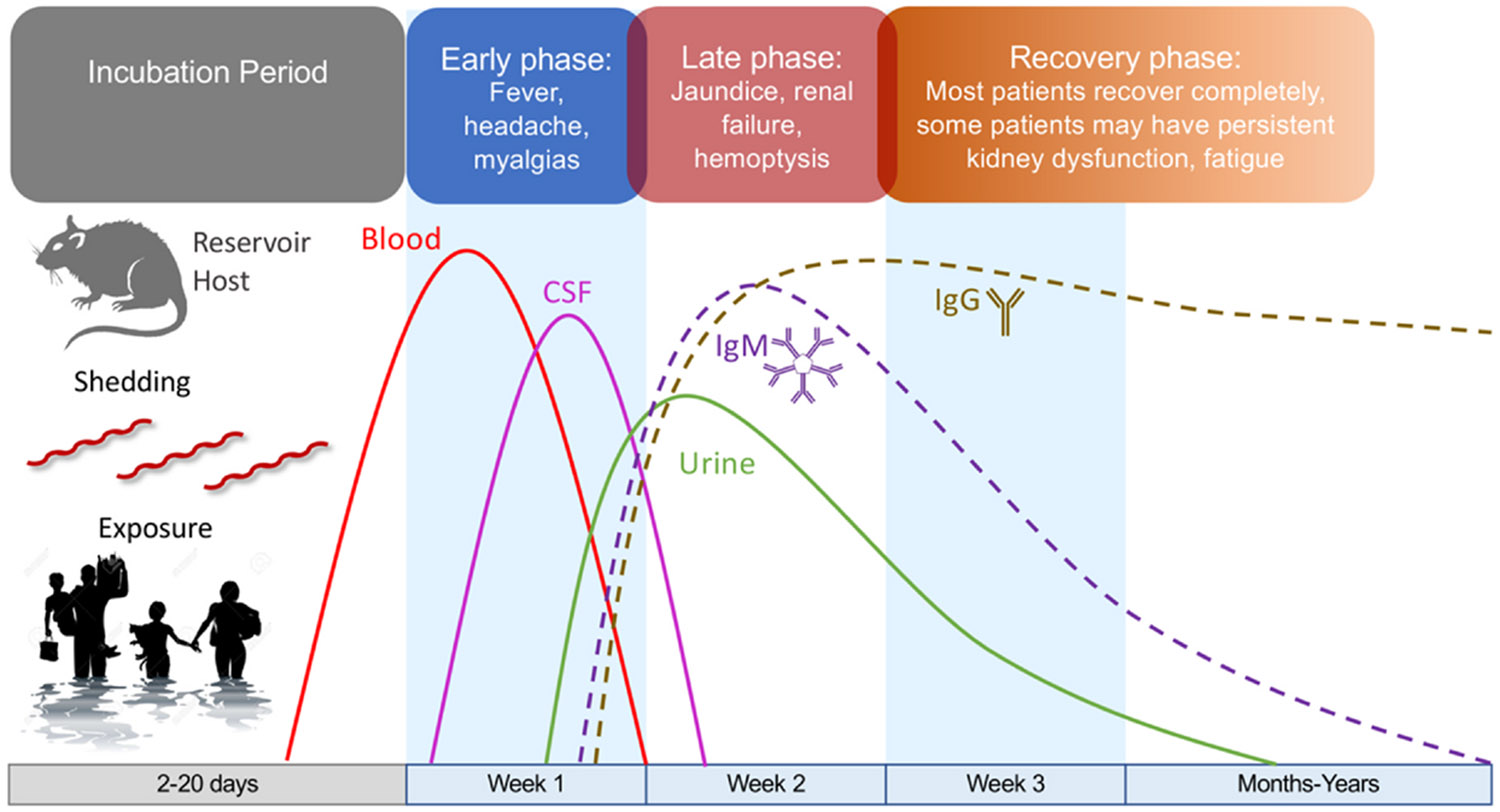
Time course of leptospirosis illustrating pathogen dynamics, immune response, and clinical progression from exposure to recovery. Following exposure to contaminated environments and a 2–20 day incubation period, *Leptospira* disseminate via the bloodstream, reaching high concentrations during the early phase (fever, headache, myalgias). This stage offers a narrow but critical therapeutic window—typically within the first week after symptom onset—when antibiotic treatment is most effective at preventing progression to severe disease. Without early intervention, patients may enter the late phase (Week 2), marked by jaundice, renal failure, and/or pulmonary hemorrhage, when mortality risk is highest and treatment efficacy declines. IgM antibodies typically appear near the end of the first week, coinciding with a decline in bacteremia but not necessarily averting organ injury. IgG develops later, supporting convalescence. In the recovery phase, most patients recover completely, though some may experience persistent kidney dysfunction or fatigue for months to years. The figure highlights the urgency of rapid diagnosis and treatment in the brief interval between early symptom onset and irreversible organ damage.

Early diagnosis and treatment of leptospirosis is best understood within the framework of the disease’s temporal progression (Fig. 1). Following exposure, infection with pathogenic *Leptospira* species enters an incubation period—typically lasting 2 to 14 days—during which the bacteria spread throughout the body and replicate un-detected. This incubation phase is subclinical, occurring beneath the threshold of immune activation and perception of clinical symptomatology. Emerging evidence suggests that pathogenic *Leptospira* species employ a number of sophisticated strategies to evade and suppress early detection by the innate immune system, which enable silent dissemination and proliferation. Organisms frequently achieve high bloodstream concentrations of 10^4^–10^6^ per milliliter of blood - the height of the leptospiremia is correlated with subsequent clinical severity. Once immune recognition finally occurs, there is a transition into the acute symptomatic phase, typically marked by fever, chills, headache, myalgias, fatigue, malaise, and other modes of clinical presentation, which are nonspecific and vary among patients. The transition from the pre-clinical to the acute phase may be abrupt, depending on burden of infection and the pace of immune system activation. Within approximately 3 to 7 days after symptom onset, IgM antibody formation begins, eventually resulting in clearance of organisms from the bloodstream. Antibody opsonization of viable organisms triggers a surge in leptospiral antigen presentation to phagocytes and other elements of the reticuloendothelial system, triggering a systemic and local host response resulting in damage to multiple organ systems—including the kidneys, liver, lungs, heart, and central nervous system. The relatively short window for early, effective antibiotic intervention lies in the period between symptom onset and antibody-mediated clearance of organisms from bloodstream and tissues. Timely recognition and treatment during this window are critical to reducing clinical impacts and preventing serious complications.

**Fig. 2.**
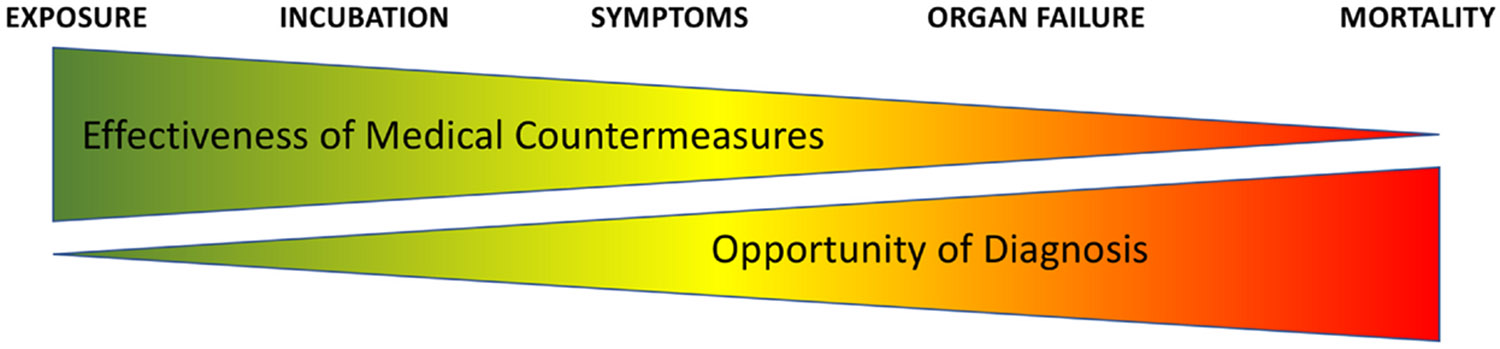
Tradeoffs between diagnostic sensitivity and therapeutic efficacy. Inverse relationship between the effectiveness of medical countermeasures and the opportunity for diagnosis in leptospirosis. In the earliest stages—soon after exposure and at the onset of symptoms—antibiotics and supportive care have the greatest potential to prevent progression to organ failure and death. However, diagnosis at this stage is challenging because current tools often lack the sensitivity to detect infection before seroconversion, when symptoms are still nonspecific. As disease advances, characteristic clinical signs make diagnosis easier, but the effectiveness of treatment declines sharply once organ damage has occurred. This gap highlights the urgent need for highly sensitive, accurate diagnostics that can identify leptospirosis during early infection, enabling timely intervention and maximizing patient survival.

The topic of early diagnosis and treatment of leptospirosis has received relatively little attention in the medical literature, in part because of the unavailability of rapid, point-of-care diagnostic tests. Clinical diagnosis, including various scoring systems involving modifications of Faine’s criteria has been appropriately emphasized. However, clinical diagnostic approaches are limited by the nonspecific and variable ways that leptospirosis presents. In the absence of patient and physician awareness, leptospirosis is frequently missed and confused with other causes of acute febrile illness. Point-of-care serologic testing, such as lateral flow platforms, may be negative at an early stage when antibiotics are most effective. Patients at risk of leptospirosis have limited access to healthcare and may have to travel long distances to seek medical attention. They may choose not to do so at an early stage either because they assume that clinical or laboratory diagnosis and treatment would not be effective or because they don’t recognize the potential seriousness of their condition. As a result, patients often present with jaundice, renal insufficiency, and other late manifestations of leptospirosis, at which point diagnosis is more straight-forward but treatment is less effective (Fig. 2). A shift in perception is needed to place leptospirosis in the context of a general understanding of infectious diseases, which is that early diagnosis and treatment leads to better healthcare outcomes as it is more efficient and lowers the overall cost of care than late treatment requiring hospitalization or even intensive care. Development of better, faster, less expensive diagnostic tools for leptospirosis is essential to enhance clinical decision-making, enable timely treatment, and reduce the overall burden of the disease—especially in underserved and vulnerable populations.

## Clinical diagnosis

Given the settings in which leptospirosis typically occurs, clinicians have relied primarily on epidemiology of exposure, rainfall, and flooding and patient signs and symptoms to diagnose leptospirosis, which are summarized in Table 1. Fever, myalgia, headache, and anorexia are some of the most frequent symptoms found in case series, particularly in patients with early infection.^7^ Given that these similar symptoms are frequently found in patients with AUFI with other etiologies such as dengue and influenza, a number of clinical scoring systems have been developed for assessing the likelihood that an acute febrile illness may be due to leptospirosis. The original Faine’s Criteria consisted of various point numbers assigned to various clinical (Part A) and epidemiologic (Part B) criteria.^8^ A score of 20–25 was considered possible leptospirosis, while a score ≥26 was presumed leptospirosis. Addition of bacteriologic or serologic testing is included in Part C for confirmation though those results are typically available at a later point in time. The original Faine’s Criteria were modified in 2004 to provide separate scores for different types of epidemiologic risk factors including rainfall, contact with a contaminated environment or an animal, which were previously lumped together, and to add scores for newer serologic testing approaches.^9^ Faine’s Criteria were modified again in 2013 as part of the Indian Guidelines for the diagnosis and management of leptospirosis to add scores for hemoptysis or dyspnea in Part A and rapid diagnostic tests in Part C.^10^

The modified Faine’s criteria were evaluated in a large study by Bandara et al. involving 168 patients admitted to hospital with suspected leptospirosis, of whom 66 patients subsequently had their diagnosis confirmed by MAT and/or PCR.^11^ The median time from onset of fever to hospital admission was 6 days and some patients had symptoms of more advanced disease, with 33% of patients having jaundice and 15% with dyspnea, indicating liver and lung involvement, respectively. Among patients with confirmed leptospirosis, sensitivity was only 17% for presumed leptospirosis and 39% for a possible or presumed diagnosis of leptospirosis. Subsequently, a New Caledonia study of a cohort of 105 patients with suspected leptospirosis of whom 35 had confirmed infection, many of whom had more severe disease (57% jaundice, 66% dyspnea or hemoptysis) found a higher sensitivity of 63% for presumed leptospirosis and 90% for possible or presumed leptospirosis. As expected, higher sensitivity for this scoring system was observed in a patient cohort with more advanced disease.

Although Faine’s Criteria was certainly useful in terms of providing a consistent case definition, its practical use in early diagnosis is not ideal. Keeping track of the scores for 13 different criteria is cumbersome in a busy clinical setting. Conjunctival suffusion, considered to be pathognomonic for leptospirosis was weighted heavily in the scoring system, particularly when combined with muscle pain and evidence of meningismus. Though highly specific, suffusion is not a particularly sensitive criterion, as suffusion is found in only 25%–60% of patients in various clinical series.^12^ Patients with early leptospirosis often fall into the category of undifferentiated febrile illness. Symptoms of headache and myalgias are often present in leptospirosis, but they are also frequently present in other febrile illnesses, so emphasizing such symptoms in a scoring system doesn’t contribute much to specificity. Interestingly, the original criteria published in 1982 called for a more specific symptom of “headache of sudden onset”,^8^ though subsequent evaluations of the criteria left out “of sudden onset” so it’s unclear how consistently this occurs in leptospirosis nor how often that modifier was applied in clinical studies.

There are a number of problems with the development and evaluation of scoring systems based purely on clinical criteria. The original process of selecting the symptoms to be included and what scores should be applied in Faine’s Criteria and its modifications may have been fairly empiric. In addition, clinically-based scoring systems fail to take advantage of more objective criteria available through routine laboratory tests, which are available with a short turnaround time in many clinical settings. Patients with leptospirosis often present with a number of abnormalities on routine laboratory testing such as electrolyte abnormalities including hypokalemia and hyponatremia, mild to moderate elevations in liver transaminases and direct (conjugated) bilirubin, neutrophilia, anemia, and thrombocytopenia, depending on the level of severity and organ dysfunction. However, it had been unclear to what extent such laboratory abnormalities might enhance the sensitivity and specificity to diagnosis of leptospirosis and differentiation from other causes of acute febrile illness.

For this reason, a Sri Lankan team of Rajapakse et al. took a more systematic approach to developing a diagnostic scoring system in a study of 592 febrile patients of whom 232 patients had leptospirosis confirmed by MAT.^13^ In this study, there were 450 patients in the derivation cohort and 142 patients in the validation cohort. Although time from onset of symptoms to study entry was not described, patients with confirmed leptospirosis had evidence of more advanced disease given that 41% had elevated bilirubin and 58% of patients went on to require hemodialysis; 3% of patients died. Specific cutoffs for bilirubin (> 30), serum creatinine (> 150), neutrophilia (> 80%), and thrombocytopenia (< 85,000) were established and assigned scores based on the ability of each criterion to differentiate patients with leptospirosis from those with non-leptospirosis fever. Interestingly, a history of “exposure to potentially contaminated water or soil” had the strongest positive association and was therefore assigned the highest score. In this validation cohort, they assessed how different total scores would have performed and in the optimal scenario; a score of 14 achieved a sensitivity of 80% and specificity of 60%. Unfortunately, they did not compare their results with how Faine’s criteria would have performed in their study population, so it’s difficult to know to what extent inclusion of routine laboratory test results and a more systematic approach improved diagnostic accuracy.

A subsequent study performed in Thailand also took a systematic approach to develop a diagnostic scoring system for leptospirosis.^14^ They enrolled 221 patients presenting at a participating medical center with clinical suspicion of leptospirosis, 50% of whom had confirmed infection based on MAT (≥1:400 or 4-fold rise in titer), culture, and/or PCR. Among the leptospirosis patients in this study, 32.7% had clinical jaundice, 13.5% had dyspnea, 35.6% had evidence of acute kidney injury (AKI), and the mean time from onset of fever to enrollment was 4 days. Multivariable regression analysis was performed to identify predictors that would contribute to diagnostic accuracy. Scores were assigned based on the resulting odds ratios. Significant predictors of leptospirosis were the combination of hypokalemia and hyponatremia, hypotension, anemia, muscle pain, ≥80% neutrophilia with a WBC < 10,000/μL, jaundice, AKI, and pulmonary opacity. Two diagnostic score models were created and tested on an independent validation cohort, with sensitivity of 73.5% and specificity of 73.7% in the simplified model. This approach had several advantages over previous studies, including a much higher positive predictive value (indicating fewer false positives) and the use of both serology and molecular testing to confirm positive cases. Addition of molecular testing was key, as many patients with early leptospirosis are seronegative.^15^

In an effort to develop a more simplified scoring system for diagnosis of early leptospirosis, researchers in Thailand developed an OPD scoring system optimized for use in the outpatient department (OPD) setting.^16^ Their study population consisted of 260 suspected leptospirosis patients presenting in community healthcare settings, of whom 82 had confirmed leptospirosis. In this cohort, leptospirosis patients had relatively early infection – the mean time from onset of fever was 3.0 days, only 6% had clinical evidence of jaundice and 17% had evidence of dyspnea. Only 7% of patients eventually required a higher level of care and there were no fatalities. Multivariable odds ratios were calculated for a variety of signs, symptoms, exposure types, and routine laboratory test results including urinalysis. Interestingly, no clinical signs or symptoms were found to provide significant diagnostic value. In contrast, exposure to a body of water potentially contaminated by animals and especially wet ground at a workplace, indicating exposure for an extended period of time, had significant diagnostic utility. Urinary blood and/or protein on a simple urine dipstick test were found to be useful, which is consistent with the renal tropism of leptospirosis. As in previous studies, a blood count with a differential showing ≥80% neutrophilia was helpful. Scores for each of these five criteria were assigned weights based on the strength of their odds ratios and results with different total scores were compared: A total score of ≥3.5 gave a sensitivity of 72.4% and specificity of 61.7%. Confirming the utility of this OPD scoring approach for early leptospirosis, only 3% of leptospirosis patients in their cohort achieved a score of ≥26 for presumed leptospirosis using the original Faine’s criteria scoring system.

In summary, a number of clinical scoring systems with and without routine laboratory testing criteria have been developed to aid in the rapid diagnosis of leptospirosis among patients with acute febrile illness. Faine’s Criteria served as an early framework that evolved over time to incorporate new clinical, epidemiologic, and diagnostic information. Evaluations in Sri Lanka and New Caledonia revealed variable sensitivity, with better performance among patients with more advanced disease. The complexity and limited early sensitivity of Faine’s system prompted development of more data-driven alternatives. Studies in Sri Lanka and Thailand applied systematic approaches using multivariable analyses to identify predictive clinical and laboratory features, including jaundice, AKI, neutrophilia, and environmental exposures. These newer models, particularly one tailored for outpatient settings, demonstrated improved sensitivity for early disease and highlighted the diagnostic value of simple tests like urinalysis and CBC, especially when combined with exposure history. Smartphone-based clinical diagnostic decision support systems, such as the ESIDA app, may be able to facilitate use by frontline physicians.^17^ These updated tools offer practical advantages in early diagnosis, especially in resource-limited or primary care settings.

## Diagnostic biomarkers

A growing body of evidence supports the potential role of protein and molecular biomarkers based on host response to assist in identification of an infectious pathogen.^18^ Such biomarkers, applied either individually or in combination, have the potential of providing diagnostic insights in the acute care setting that are both broadly applicable and potentially provide insights into the host response that are superior to serology and molecular diagnostics. The most well studied biomarker in leptospirosis is C-reactive protein (CRP), which has been found to be useful in differentiating bacterial infections such as leptospirosis from dengue and other viral infections.^19-24^ For example, two relatively large studies found that CRP levels > 50 mg/L were highly sensitive for leptospirosis at both early and late stages of infection and were helpful in differentiating leptospirosis from dengue fever.^19,20^ An advantage of CRP is that levels can be obtained rapidly (~4 min) from a fingerstick using commercial testing platforms.^25^

CRP is one member of a class of classic acute phase reactants produced by hepatocytes in response to inflammatory cytokines including IL-1β and IL-6. Additional acute phase reactants with diagnostic potential were identified when mass spectroscopy was performed on plasma from patients with leptospirosis vs dengue vs healthy controls.^23^ In addition to CRP, three additional classic acute phase reactants were consistently found in higher amounts in leptospirosis patients: leucine-rich alpha-2-glycoprotein 1 (LRG1), lipopolysaccharide-binding protein (LBP), and serum amyloid A-1 (SAA1). Interestingly, all four acute phase reactants were differentially expressed both in early leptospirosis, when patients were PCR+ and seronegative, and later when patients were PCR− and seropositive. This same mass spectroscopy study also found that leptospirosis patients at both early and later stages had lower plasma levels of several platelet alpha-granule proteins (see Table 2 for details) than found in healthy controls but higher than in dengue fever patients. Although procalcitonin is not a classic hepatic-produced acute phase reactant in that it is produced in extra-hepatic tissues, it has been found to be useful as a diagnostic biomarker for differentiating leptospirosis from dengue fever.^24^

In contrast to the proteomic studies, reports available to date indicate that although the levels of some cytokines differ between leptospirosis and dengue, most cytokines are not particularly useful diagnostically in differentiating leptospirosis from other causes of AUFI.^26,27^ In 2014, Conroy et al. published a study surveying cytokine levels in patients with either leptospirosis or dengue and reported that IL-18 binding protein (IL-18BP) and soluble endoglin (sENG) were differentially expressed.^26^ However, a subsequent 2015 study by Michels et al. found that IL-18BP levels were comparable in both infections.^27^ The differences in the results between these two studies may be explainable by the indication that the leptospirosis cohort in the 2014 appeared to have relatively mild disease - very few of the leptospirosis patients were febrile. This is a reminder that in interpreting such studies, it is important to keep in mind that the host response to leptospirosis differs depending on the stage and severity of disease and that it evolves over time, such that the early host response during the leptospiremic phase may be quite different from the later host response after antibody formation (Fig. 1). Another important caveat is that although CRP and other protein biomarkers are helpful in distinguishing leptospirosis from dengue, high CRPs can be found in a number of relevant bacterial infections, including typhoid, rickettsial infections, sepsis, brucellosis, and melioidosis, and parasitic infections including malaria and visceral leishmaniasis.

A host response signature composed of multiple transcriptional targets has been developed that is able to differentiate viral from non-viral infectious etiologies of acute febrile illness occurring globally, including leptospirosis.^28^ For such an approach to be relevant in a clinical setting, the signature would need to be ported to a multiplex assay qRT-PCR assay platform. A rapid (~1 h) transcriptional host response test has been described that can be performed on portable workstation for discrimination of bacterial vs viral infections.^29^ Positive identification of a bacterial etiology would certainly be useful given that a range of antibiotics are effective for leptospirosis. Nevertheless, discrimination between different bacterial etiologies would certainly have even greater clinical and epidemiologic value.

## Leptospirosis-specific testing

Leptospirosis-specific testing methods including culture, serology, and molecular testing are desirable because they can provide improved diagnostic certainty to help focus appropriate patient management. Culture remains a valuable approach for epidemiologic reasons and culture-based methods have improved dramatically in recent years.^30^ However, even under ideal growth conditions, the organism’s replication rate is too slow to facilitate a timely diagnosis, so these approaches will not be further evaluated here. Serological tests are designed to detect antibodies recognizing *Leptospira*-specific antigens. The time at which the earliest IgM antibodies begin to be detectable is variable among patients. Typically, this occurs within 3–7 days after onset of symptoms, so serological tests may be negative when patients first present for medical attention.^31^ PCR and other molecular approaches for direct detection of leptospiral DNA in a clinical sample are the most sensitive tests in early infection. Molecular approaches are particularly valuable for identifying patients that may benefit from early antimicrobial therapy and for this reason will be given more attention in this brief review. Readers seeking more detailed information about leptospirosis-specific testing are referred to more comprehensive reviews.^32^

Among serological tests, the reference standard continues to be the microscopic agglutination test (MAT). In this test, dilutions of patient sera are mixed with live organisms and monitored by darkfield microscopy. Advantages to MAT are its sensitivity, ability to identify serogroup-specific reactivity, and ability to measure the titer of the serological response, which allows comparison of acute and convalescent sera, which is important in endemic settings where patients may have pre-existing leptospiral antibodies. Considerable technical expertise is required to perform the MAT, cultivate the live antigens, and maintain a quality control program.^33^ As such, it is typically restricted to reference laboratories and used for confirmatory testing. Enzyme-linked immunosorbent assay (ELISA) methods involve detection of IgM antibodies by incubating patient samples in the presence of immobilized leptospiral antigens. These antigens are obtained from whole-cell lysates or detergent extracts of leptospiral strains or recombinant antigens in some cases. Leptospirosis-specific antibodies are detected with a secondary antibody linked to an enzyme that catalyzes a colorimetric reaction that can be read spectrophotometrically with an ELISA plate reader. IgM ELISA assays are generally more sensitive than MAT in early leptospirosis, as IgM antibodies often become detectable before serogroup-specific agglutinating antibodies are measurable by MAT.^34^ A number of IgM ELISA kits with a turnaround time of 2–3 h are available commercially for leptospirosis diagnosis.^35^

A number of lateral flow and other serologic rapid diagnostic tests (RDTs) are commercially available and can be performed within 15–30 min at the point-of-care with minimal training. Serologic RDTs for leptospirosis typically have lower sensitivity and specificity than ELISAs but are ideal for under-resourced settings lacking a laboratory infrastructure and can be deployed quickly in outbreak settings.^34,36^ Three commercially available rapid serologic tests were compared on 5144 samples, including samples from 367 patients with leptospirosis.^37^ Striking differences in sensitivity were found among the tests, particularly in samples obtained early after the onset of illness; the highest sensitivity was 62% and 75% in samples from patients with 0–4 and 5–10 days, respectively, post-onset of illness. A subsequent prospective diagnostic accuracy study was performed in Thailand, comparing five commercially available lateral flow IgM tests.^38^ Again, sensitivities ranged widely between tests with one of the tests exhibiting approximately 82% sensitivity on days 4–6 after fever onset. Other studies have also reported wide variations in sensitivity between RDTs.^39,40^ These findings under-score the variable and limited sensitivity among RDTs, particularly during early infection, reinforcing the need for more robust field-adaptable diagnostics.

To overcome the time gap before antibody seroconversion, rapid antigen detection tests have been explored to directly identify leptospiral components such as LipL32 (a dominant leptospiral lipo-protein), and lipopolysaccharide (LPS) in blood or urine. Several proof-of-concept studies have demonstrated the feasibility of direct antigen detection using various immunoassay platforms, including a gold nanoparticle–based immunochromatographic test using anti-LipL32 antibodies,^41^ an electrochemical lateral flow assay detecting LipL32,^42^ immunofluorescent nanotube probes targeting LipL32,^43^ and a monoclonal antibody-based immunochromatographic assay detecting LPS antigen in urine.^44^ Although these approaches confirm that circulating leptospiral antigens can be detected early in infection, antigen-based lateral flow assays are not yet clinically validated for routine human diagnosis.

Polymerase chain reaction (PCR) is the most widely used molecular diagnostic method used for the early detection of leptospirosis. PCR detects leptospiral DNA directly in clinical specimens such as whole blood, serum, plasma, cerebrospinal fluid, or urine—typically during the first 5 to 10 days of illness, when leptospiremia is present and before antibodies are detectable. Inclusion of urine PCR with blood PCR improves overall diagnostic yield and enhances early diagnostic sensitivity, particularly within the first three days after symptom onset.^45^ Conventional and real-time (quantitative) PCR assays have all been developed to target conserved leptospiral genes, such as *lipL32*, 16S *rRNA*, and *secY*. An advantage of molecular testing is its high sensitivity for leptospiremia in the early phase of illness (before seroconversion) when mean leptospiral copy numbers are typically 420 and 1440 copies/mL, respectively, in mild and severe disease, respectively.^46^ A large number of clinical studies have reported PCR sensitivities varying from 52% to 100% depending on the timing of sample collection, type of specimen, and method used.^47^ A comparison of four qPCR assays found slightly higher sensitivity in plasma than in whole blood and serum.^48^ Turnaround time is typically 1–3 h for qPCR after an initial DNA extraction step. In addition, PCR requires specialized equipment and trained personnel, and test availability may be limited outside of reference or hospital laboratories. The BioFire global fever panel is a moderately complex PCR-based test with a run time of ~1 h and has been assessed to have 94% sensitivity (15/16 positives) for leptospiral detection in a large multicenter study.^49^ Loop-mediated isothermal amplification (LAMP) is a promising, field-adaptable approach for early detection of leptospirosis, offering high diagnostic utility where PCR is un-available. A recombinase polymerase amplification–CRISPR/Cas12a (RPA-CRISPR/Cas12a) system targeting the *lipL32* gene has been developed with a lateral flow readout for point-of-care testing.^15^ Molecular tests can be combined with serologic testing for higher diagnostic accuracy in early infection.^50,51^ In a study of leptospirosis patients in Thailand, serologic or molecular testing alone achieved sensitivities of 54% and 63% respectively among patients tested within 4 days of onset of symptoms, while the combination of both approaches yielded 87% sensitivity.^51^ Given their advantages for early diagnosis, molecular methods are being increasingly incorporated into clinical practice and are especially valuable for early diagnosis. As the turnaround time improves, metagenomic sequencing may ultimately replace targeted molecular methods for diagnosis of leptospirosis, especially given the variability in clinical presentation.^52^

## Severity prediction

Early prediction of disease severity in patients with leptospirosis is critical for guiding clinical management, as those at higher risk for complications may benefit from more aggressive interventions, such as intravenous antibiotic therapy, hospitalization, intensive monitoring, fluid and electrolyte management. Recent comprehensive reviews of severity indicators in leptospirosis have underscored the importance of identifying indicators of organ dysfunction,^53-55^ aligning with the well-established observation that elevated SOFA scores are strongly associated with worse outcomes regardless of the underlying condition.^56^ Ideally, risk factors can be recognized and severity predictions made in early disease, as indicated in Fig. 2, before the onset of severe complications such as hepatorenal failure or pulmonary hemorrhage, when it is already too late to intervene to avoid such complications. For example, the simple demographic factor of a patient’s age has emerged as an important leptospirosis mortality risk factor in a number of studies.^1,57-63^ Other demographic factors associated with worse outcomes include alcoholism and cigarette smoking.^58-60,64^

In the initial evaluation of a patient with suspected or confirmed leptospirosis, dyspnea and other respiratory symptoms are the most consistently identified predictors of severe disease.^55^ Other worrisome clinical signs suggesting that a patient may be at risk for severe disease or death include hypotension, dyspnea, tachycardia, oliguria, jaundice, bleeding, and altered mental status.^1,55,57-59,62,64-67^ Laboratory findings that can be obtained rapidly in many clinical settings may also be useful include a complete blood count looking for anemia, leukocytosis, and thrombocytopenia, serum creatinine, bilirubin, lactate, C-reactive protein (CRP), and procalcitonin.^55,64-66,68^ Electrocardiographic changes have also been found to be a marker of severity.^67,69^ In the study by Mikulski et al., an elevated Simplified Acute Physiology Score (SAPS) II score was found to be predictive of severe disease.^65^ SAPS II scores are calculated from 12 physiologic variables based on patient age, vital signs, the Glasgow Coma Scale, blood chemistries, blood count, oxygenation, and urine output. Additional laboratory markers that may be useful include the plasma neutrophil gelatinase-associated lipocalin (pNGAL) and long pentraxin (PTX3), a serum acute phase reactant with a structure similar to CRP.^46,65,70^ When measured in early infection, the leptospiral burden in the blood as measured by qPCR is an important severity risk factor.^46,64^ In a survey of 92 inflammation-related plasma proteins in patients with early leptospirosis, a reduction in the neutrophil chemotactic protein CXCL5 was the strongest predictor of worse clinical outcomes.^71^ The combination of CXCL5, albumin, bicarbonate, and lymphocyte percentage improved prognostic accuracy (AUC 0.86) compared to CXCL5 alone (AUC 0.77). Among more well-known cytokines, tumor necrosis factor (TNF), interleukin-10, and IL-6 are notably elevated in severe and fatal leptospirosis.^53,71-76^ In addition, IL-8, IL-6, granulocyte-macrophage CSF (GM-CSF), vascular endothelial growth factor (VEGF), monocyte chemoattractant protein-1 (MCP-1) and interferon-inducible protein 10 (IP-10) have been associated with hemorrhage and pulmonary symptoms.^74^

Markers of oxidative stress appear to be correlated with severity in leptospirosis. Levels of reactive oxygen species were found to be higher in patients with leptospirosis than healthy controls and depletion of the antioxidant reduced glutathione (GSH) was associated with acute renal insufficiency in acute leptospirosis.^77^ Consistent with these findings, increased protein carbonyl, a biomarker of oxidative stress, has been found to be elevated in severe leptospirosis.^78^ Conditions associated with oxidative stress are well known to precipitate hemolysis in patients with glucose-6-phosphate dehydrogenase (G6PD) deficiency, and G6PD deficiency has been implicated in the pathogenesis of anemia in leptospirosis.^79^ Anemia is common in leptospirosis, and is presumably involves some combination of hemorrhage and hemolysis.^80^ However, the frequency with which oxidative stress contributes to hemolytic anemia requires further examination.

Two clinical scoring systems, SPiRO and QuickLepto, have been developed to aid in risk stratification.^63,81^ The SPiRO score is based on three readily available clinical features at presentation: oliguria (≤500 mL urine/24 h), abnormal findings on respiratory auscultation, and hypotension (systolic blood pressure ≤100 mmHg). Each variable contributes one point to a three-point score, with the likelihood of severe disease increasing sharply with higher scores—ranging from 3% at a score of 0 to 100% at a score of 3. A SPiRO score < 1 has a high negative predictive value (97%), making it a useful tool to rule out severe disease. The QuickLepto score includes five variables—age over 40, lethargy, pulmonary symptoms, mean arterial pressure < 80 mmHg, and hematocrit < 30%—and demonstrates good predictive accuracy for mortality (AUC-ROC = 0.788), outperforming both SPiRO and quick SOFA in this regard.

Transcriptomic approaches are promising not only for diagnosis but also for assessing severity risk. CHI3L1 and CCL5 (RANTES) are among candidate transcriptomic markers demonstrating differential expression in severe disease.^82^ Recent advances in host transcriptomic profiling have identified circulating microRNAs as promising biomarkers for severity prediction. In a prospective study, microtranscriptome profiling using NanoString technology revealed distinct miRNA expression signatures between severe and non-severe cases of leptospirosis. Specifically, miR-155-5p and miR-630 were significantly upregulated in severe disease and, when combined with serum bicarbonate, showed good discriminatory performance (AUC = 0.79).^83^ In a subsequent validation study, serum miR-601 and miR-630 were found to be elevated in patients with icteric leptospirosis and were predictive of subsequent acute liver failure and poor survival outcomes.^84^ More recently, a multi-omics study focusing on severe pulmonary hemorrhagic syndrome (SPHS) identified miR-5010-3p and miR-147b-3p as significantly associated with SPHS, with AUCs of 0.76 and 0.70, respectively. When combined with other miRNAs (miR-548ai and miR-224-5p), predictive accuracy increased (AUC = 0.86), and the miRNA panel further improved performance of an existing clinical model.^85^ Integrated pathway analyses implicated TNF signaling as a key mechanism underlying severe pulmonary complications. Together, these findings support the utility of microRNA-based biomarkers for early severity stratification and highlight the potential of multi-omics approaches to uncover key host response pathways in leptospirosis.

## Impact of early treatment on healthcare outcomes

When diagnosed early, leptospirosis can be effectively treated with a range of low-cost antibiotics. Early treatment with antibiotics is a critical factor in preventing the progression of leptospirosis to severe disease, including organ failure. Likewise, a delay in treatment is a risk factor for more severe outcomes.^64,86,87^ Antibiotics such as doxycycline and penicillin are highly effective when administered in the early stages of infection. Educating healthcare providers on the importance of initiating antibiotics early when there is a high index of suspicion, with or without availability of confirmatory diagnostic test results, is key to improving outcomes in leptospirosis.

Treatment with antibiotics is effective in early disease and there are no reports of antibiotic resistance in *Leptospira* species. Doxycycline is considered first-line treatment for mild-moderate suspected cases, with penicillin (IV) recommended for severe cases (i.e. organ failure) or in pregnant women.^12^ Both antibiotics reduce the severity and duration of the disease, with doxycycline often preferred due to its broad availability and ease of administration. Azithromycin, dosed orally, is also an alternative for patients unable to tolerate doxycycline or penicillin,^12^ with additional medications such as tetracycline, ceftriaxone, and cefotaxime occasionally used in specific clinical scenarios. Each one of these treatments is included on the WHO essential medicines list, demonstrating the widespread availability of effective therapy (https://www.who.int/publications/i/item/WHO-MHP-HPS-EML-2023.02).

Multiple studies have demonstrated that initiation of antibiotics before the onset of severe organ dysfunction significantly enhances recovery and overall morbidity.^26-31^ The ideal timeframe for initiation of therapy is within the first six days of symptom onset, with greatest reductions in fever duration and related complications occurring when antibiotic treatment is initiated within two days of symptom onset.^88-93^ Pioneering research into this involved a randomized, placebo-controlled trial conducted at a US Army jungle training center in Panama,^88^ where McClain et al. found that doxycycline 100 mg BID initiated, on average, two days after symptom onset and prior to the onset of renal failure or jaundice, reduced post-antibiotic fever duration from 5.4 days to 3.7 days and, most critically, prevented the onset of renal failure or jaundice.^88^ As shown in Table 1, six additional randomized controlled trials, involving almost 300 patients with confirmed leptospirosis, demonstrate significantly earlier resolution of fever and other clinical parameters when either penicillin, ampicillin, or oxytetracycline was initiated (average treatment onset 6 days after onset of symptoms).

Another example is the study by Fairburn and Semple, which reported a 1.7 day reduction in the duration of post-antibiotic symptoms (from 7.7 days to 6.0 days) in patients with leptospirosis treated with penicillin.^89^ Although this difference was not reported as significant, a Mann-Whitney test of the data provided in their paper showed the difference in fever duration to be significant (p < 0.02). In particular, penicillin therapy reduced the percentage of patients with greater than one week of fever from 45.2% to 9.5%. Importantly, the majority of studies reporting antibiotic efficacy involved early leptospirosis with study populations in whom a majority of patients had not yet developed significant organ dysfunction. The conclusion that antibiotics may also have benefits at later stages is based on studies by Marotto et al.^31^ and Watt et al.,^94^ which demonstrated shorter duration of fever in patients treated with antibiotics even though 70%–73% had jaundice at the time of treatment. In contrast, reports that failed to show an impact of antibiotics involved patient populations consisting almost entirely of patients with late leptospirosis and advanced organ dysfunction,^95,96^ where response to antibiotics would be highly unlikely. These results are not surprising given the association of early antibiotics with improved outcomes is a widely recognized therapeutic principle, common to many types of infectious diseases.

Therapeutic interventions for leptospirosis are not limited to antibiotics. Proper attention to hydration and electrolyte balance is beneficial at the stage of non-oliguric renal dysfunction, and other types of supportive care play crucial roles in management of more severe cases of leptospirosis.^12,97^ In patients who meet appropriate clinical criteria, initiation of peritoneal dialysis, continuous or intermittent hemodialysis is clearly indicated.^98^ For patients with advanced AKI, early initiation of renal replacement therapy using peritoneal dialysis (PD), intermittent hemodialysis (IHD), or continuous renal replacement therapy (CRRT) should be guided by clinical status and resource availability. A recent multicenter randomized controlled trial^99^ found no significant difference in 28-day mortality or dialysis-free survival between lower-dosage acute PD and IHD. PD was associated with fewer episodes of intradialytic hypotension, supporting its use as a feasible alternative to IHD, particularly in resource-limited or hemodynamically unstable patients.

In the case of severe pulmonary hemorrhage syndrome (SPHS), a feared complication of leptospirosis with a mortality rate as high as 50%, patients may respond to aggressive treatments such as extracorporeal membrane oxygenation (ECMO).^100^ Hemoperfusion has emerged as a promising adjunctive therapy in severe leptospirosis, particularly in patients with multiorgan failure and features of cytokine storm. The technique involves the use of neutral macroporous resin-adsorbing beads to remove circulating cytokines and inflammatory mediators, contributing to hemodynamic stabilization and endothelial protection. In a recent single-center randomized controlled trial involving leptospirosis patients with renal failure and shock, adjunctive hemoperfusion led to a 36.8% reduction in 28-day mortality and showed significant improvements in serum creatinine, procalcitonin, IL-6, lactate levels, and PaO_2_/FiO_2_ ratio, even in patients also receiving ECMO support.^101^ These findings build on earlier observational work by Danguilan et al.,^102^ which incorporated hemoperfusion into a multimodal approach alongside corticosteroids and immunosuppressants. Together, these studies suggest that hemoperfusion may represent a safe and effective component of therapy in patients with severe, reversible multiorgan dysfunction due to leptospirosis.

## Implementation strategies for early diagnosis and treatment

Reducing morbidity and mortality from leptospirosis requires proactive implementation strategies that enable early recognition and timely management, particularly in resource-limited and endemic regions. Increasing awareness among both healthcare providers and the general public is a critical first step. Educational initiatives that focus on symptom recognition and risk assessment, including host susceptibility factors can support timely clinical suspicion and early intervention.

Strengthening integrated public health systems, including surveillance and referral networks, is essential for translating early suspicion into actionable diagnosis and treatment. A One Health approach that connects human, animal, and environmental health sectors enhances outbreak detection and rapid case reporting in high-risk settings. Expanding access to early diagnostic tools, such as point-of-care tests, clinical risk scores like the THAI-LEPTO score, and molecular diagnostics, is crucial for frontline decision-making. These tools should be integrated into structured care pathways supported by telemedicine consultation and timely referral mechanisms.

**Fig. 3.**
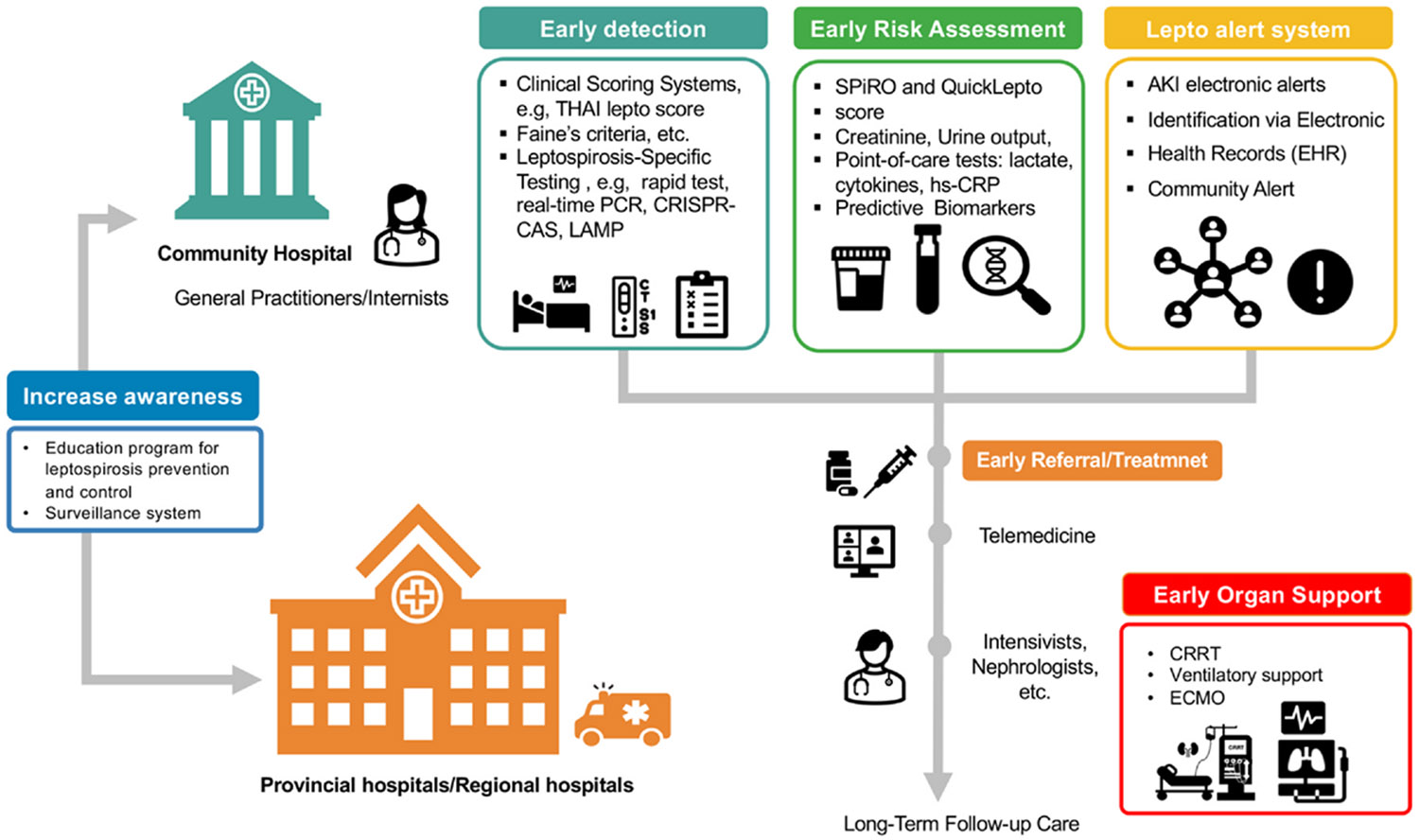
Integrated framework for improving leptospirosis outcomes through coordinated early detection, risk assessment, awareness, referral, and supportive care. At the community hospital level, general practitioners and internists utilize early detection tools—including clinical scoring systems (e.g., THAI Lepto score, Faine’s criteria) and leptospirosis-specific diagnostics (rapid tests, real-time PCR, CRISPR-Cas, LAMP)—to identify cases promptly. Early risk assessment incorporates severity prediction scores (SPiRO, QuickLepto), key laboratory parameters (creatinine, urine output, lactate, cytokines, hs-CRP), and predictive biomarkers to guide triage. A lepto alert system, leveraging electronic health record alerts, acute kidney injury (AKI) notifications, and community alerts, facilitates rapid recognition and response. Awareness programs and surveillance systems support prevention, early care-seeking, and timely diagnosis. Early referral and treatment, supported by telemedicine consultation with specialists, ensures rapid escalation of care to provincial or regional hospitals. For patients with severe disease, early organ support—including continuous renal replacement therapy (CRRT), ventilatory support, and extracorporeal membrane oxygenation (ECMO)—is initiated. This integrated approach, from community-level detection to advanced hospital-based interventions, is designed to maximize timely treatment, prevent disease progression, and improve survival and long-term outcomes in leptospirosis patients.

Fig. 3 presents a comprehensive framework for improving early detection and management of leptospirosis in endemic areas. At the community hospital level, early diagnosis is facilitated by the use of clinical scoring systems, leptospirosis-specific rapid tests, creatinine and urine output monitoring, and electronic health record alerts. General practitioners evaluate patients and determine the need for referral or care bundle initiation. Telemedicine links enable remote consultation with nephrologists to support early triage decisions. Patients requiring advanced care are referred to regional hospitals where nephrologists provide organ support such as dialysis, ventilation, or ECMO. Long-term follow-up is emphasized to monitor for chronic kidney disease. The model also includes public education and community surveillance to promote early care-seeking and case detection.

Finally, investment in diagnostic infrastructure, clinical training, and care delivery systems must be matched by policy recognition of leptospirosis as a neglected tropical disease. This would help mobilize resources and promote sustainability of early intervention strategies in high-burden regions.

## Conclusions and call to action

Leptospirosis causes over 1 million cases and nearly 60,000 deaths annually yet remains underdiagnosed due to nonspecific symptoms and limited rapid diagnostic tools. Early diagnosis and prompt antibiotic treatment—ideally within the first days of illness—are highly effective at preventing severe complications such as organ failure and pulmonary hemorrhage. Current approaches rely on clinical suspicion, exposure history, and routine laboratory tests, supported by serologic and molecular diagnostics. While PCR offers the best early sensitivity, most molecular platforms are not yet field-deployable. Point-of-care tests, though rapid, often miss early disease if the sample is obtained before an antibody response has occurred. Emerging tools—such as LAMP assays, RPA-CRISPR/Cas12a-based diagnostics, and host-response biomarkers—offer promise but require further development for real-world, resource-limited settings.

To change the current paradigm, investment is urgently needed in more affordable, field-adaptable molecular and biomarker-based diagnostics that detect infection before seroconversion. Integrated early intervention strategies are needed, including clinical scoring systems, provider training, and public health awareness. Looking forward, policy recognition of leptospirosis as a neglected tropical disease will be essential to drive funding and implementation. Governments, funders, and global health agencies must prioritize research, infrastructure, and coordinated One Health surveillance to enable earlier diagnosis and treatment. With better tools and systems, most severe outcomes from leptospirosis are preventable — reducing the impact of chronic disease and saving many thousands of lives each year.

## Figures and Tables

**Table 1:** Approaches for early diagnosis of leptospirosis in patients with acute undifferentiated febrile illness (AUFI).^a^

Epidemiologic Factors
• Exposure to potentially contaminated water or soil – particularly wet ground at workplace and/or contact with animal water reservoir^13,16,103^
• Recent heavy precipitation, especially if there has been exposure to flooding^10,103^
Clinical – fever usually present but is not diagnostic
• Hypotension – useful in the emergency department setting^14^
• Headache - particularly when onset sudden and severe^103^
• Conjunctival suffusion, meningismus, myalgia – especially when all three present^14,103^
• Jaundice – may not be present in early disease^11,14^
• Hemoptysis and/or dyspnea^103^
Laboratory Studies
• Anemia with Hgb < 12 g/dL^14^
• Neutrophilia ≥80% - helpful in differentiating bacterial from viral infections^13,14,16^
• Thrombocytopenia < 85,000^13^
• Hypokalemia and hyponatremia – K < 3.5 & Na < 135^14^
• Organ dysfunction: Acute kidney injury, elevated bilirubin, hypoxia^11,13,14^
• Urine dipstick positive for blood or protein – renal tropism of leptospirosis^11,16,103^
Scoring Systems
• Faine’s criteria –clinical data, epidemiologic factors, culture isolation, and serology^8^
• Modified Faine’s criteria (2012) – respiratory symptoms, rainfall, PCR added^10^
• Resource Limited Setting Model – exposure plus four lab results^13^
• THAI-LEPTO score – for early diagnosis of severe leptospirosis^14^
• Outpatient Department (OPD) Score – for diagnosis in the outpatient setting^16^
Biomarkers
• Acute phase reactants: CRP, LRG1, LBP, SAA1^23^
• Platelet α-granule proteins: PPBP, PF4, THBS1^23^
• Procalcitonin^24^
• Transcriptomic signature^28^
Leptospirosis-specific testing – culture is important but slow
• PCR and other molecular tests of blood, plasma, and urine
• Rapid antigen detection – lateral flow assays for LipL32 and LPS in blood and urine
• Serology – patients may be seronegative in early infection

a See text for details.

**Table 2:** Summary of controlled studies of antibiotic therapy for leptospirosis ranked by percentage of patients with organ dysfunction.

Publication	Study design	Study size	Pre-treatment fever duration	Organ dysfunction	Summary of findings
McClain et al. *Ann Intern Med,* 1984	Double-blind, placebo-controlled RCT	placebo: 15 doxy: 14	1.9 days	J: 0% AKI: 0%	Post-treatment duration of illness, fever, headache, myalgias, and malaise significantly shorter in doxycycline group (p ≤ 0.05)
Doherty *Australas Ann Med* 1956	Retrospective comparative study	low pcn: 25 med pcn: 20 high pcn: 26	low dose: 2.0 d med dose: 2.6 d high dose: 2.5 d	J: 0% AKI: 0%	Mean fever duration reduced from 2.9 to 1.6 and 1.4 days in the low-dose, med dose, and high dose treatment groups, respectively (p < 0.002)
Fairburn & Semple *Lancet,* 1956	Sequential allocation	controls: 31 penicillin: 21	controls: 4.0 d penicillin: 4.7 d	J: 3.6% AKI: 4.8%	Mean fever duration reduced from 7.7 to 6.0 days (p ≤ 0.02)
Ross Russell *Lancet*, 1958	Consecutive alternating allocation	controls: 25 oxytet: 27	controls: 3.0 d oxytet: 3.9 d	J: 21% AKI: 42.3%	Mean fever duration reduced from 6.4 to 2.5 days and mean symptom duration from 6.9 to 4.0 days (p < 0.05)
Kocen et al. *Brit Med J,* 1962	Sequential cohort	controls: 33 penicillin: 28	early: 3.4 d late: 5.7 d	J:11.5% AKI: 68.9%	Mean fever duration reduced from 5.7 to 1.3 days and from 4.3 days to 1.4 days in early and late treatment groups, respectively (p < 0.001)
Watt et al. *Lancet,* 1988	Double-blind, placebo-controlled RCT	placebo: 19 penicillin: 23	9.0 d	J: 45.2% AKI: 76.1%	Mean fever duration reduced from 11.6 to 4.7 days (p < 0.005). Discharge by day 7: 70% in pcn group vs 23% in controls (p < 0.025)
Morotto et al. *AJTMH* 1997	Retrospective comparative study	controls: 15 pcn/amp: 28	controls: 5.4 d pcn/amp: 5.6 d	J: 69.8% AKI: 79.1%	Duration of renal dysfunction (3.6 d vs 5.3 d) and thrombocytopenia (3.9 d vs 6.5 d) was shorter in the pcn/amp group (p < 0.02)
Edwards et al. *AMTMH* 1988	RCT	controls: 41 penicillin: 38	controls: 6.7d pencillin: 6d	J: 100% AKI: 72%^a^	No differences in fever duration (6.9 vs 6.6 days) or other clinical parameters
Daher & Nogueira RIMTSP 2000	Comparative cohort study	controls: 19 penicillin: 16	controls: 7.4 d penicillin: 7.1 d	J: 100% AKI: 100%	No differences in fever duration (3 vs 2 days), duration of hospitalization (12 vs 11 days), or other clinical parameters

Abbreviations: AKI, Acute kidney injury; RCT, randomized controlled trial; pcn, penicillin; d, days; doxy, doxycycline; oxytet, oxytetracycline.

a Calculated based on mean and standard deviation of serum creatinine.
